# Natural Language Processing of Electronic Patient Records to Predict Psychiatric Inpatients at Risk of Early Readmission to Hospital Using Predictive Models Derived Through Machine Learning

**DOI:** 10.1192/bjo.2022.87

**Published:** 2022-06-20

**Authors:** Tarif Kapadi, Saturnino Luz

**Affiliations:** 1 Forward Thinking Birmingham, Birmingham Women's and Children's NHS Foundation Trust, Birmingham, United Kingdom; 2 Usher Institute, University of Edinburgh, Edinburgh, United Kingdom

## Abstract

**Aims:**

Psychiatric readmissions cause a burden on the healthcare system, incur a monetary cost and cause additional distress to acutely unwell patients. This project explores the use of the free-text of electronic patient records to predict inpatients in psychiatric hospitals at risk of readmission using predictive models generated by machine learning.

**Methods:**

Free-text was extracted from the electronic patient records of patients admitted to hospitals in Birmingham and Solihull Mental Health Foundation Trust (BSMHFT) during the five years 2015–2019 inclusive. The anonymised records were obtained via the CRIS (Clinical Record Interactive Search) database. A total of 17208 records were extracted.

The free-text entered by clinicians during an admission was extracted and processed using techniques of natural language processing to generate input vectors suitable to be used with machine learning algorithms. tf-idf (term frequency-inverse document frequency) vectors were used.

A selection of algorithms were used to train predictive models. Two-thirds of the records were used as training data with the remainder as test data. Baseline model performance was assessed and then best-performing candidates underwent hyperparameter optimisation using five-fold cross-validation to improve performance. Bayesian optimisation was used to automate hyperparameter tuning during cross-validation. Hyperparameters were optimised on the log loss function. As the dataset was imbalanced with negative instances outnumbering positive instances to a significant degree, various techniques such as random undersampling of negative instances in the training data were used to deal with class imbalance throughout this process. Following cross-validation, the best-performing models underwent performance analysis.

Models were used to make predictions on the test data. Performance was assessed using F1-measures, precision-recall curves and the average precision metric (equivalent to area under the precision-recall curve). These metrics were chosen due to their suitability in assessing models trained on imbalanced datasets.

**Results:**

The best F1 score obtained was 0.233 using a Random Forest model trained using unigram tf-idf vectors of 500 token dimension.

The best average precision obtained was 0.157 using a Support Vector Machine trained using unigram tf-idf vectors of 2000 token dimension.

Both the above results required the use of random oversampling of positive instances to improve performance on the imbalanced dataset.

**Conclusion:**

The performance indicates that the models generated are unlikely to have significant practical utility. Nevertheless, this exploratory project has produced a processed dataset with knowledge about its characteristics. This could be used for the further development of models using more complex techniques such as language modelling using neural networks.

